# Infection Risk in Biological Disease-Modifying Anti-rheumatic Drugs

**DOI:** 10.7759/cureus.80634

**Published:** 2025-03-15

**Authors:** Mourushi Isa, Ma. Rosario Rufina Ramos, Shahed Kamal

**Affiliations:** 1 General Medicine, Northern Health, Melbourne, AUS; 2 Medicine, Ateneo School of Medicine and Public Health, Pasig, PHL

**Keywords:** bdmards, biologics, immunization, infection, vaccination

## Abstract

Rheumatology patients on biological disease-modifying anti-rheumatic drugs (bDMARDs) have been proposed to be at a higher risk of infections. Our review summarizes the current evidence behind this theory as well as explores which factors predispose patients to various infections, which agents are more likely to cause infections, and which infections are common in these patients. We also aim to explore updated guidelines on infection prevention in patients on bDMARDs.

## Introduction and background

The landscape of treatment for different autoimmune conditions has changed significantly over the last few decades. In recent times, it incorporates several treatment options, including disease-modifying anti-rheumatic drugs (DMARDs) be it conventional, synthetic, or biological [1]. Although these treatment options may help control the disease, there has been concern about increased infection risk associated with the use of such medications. While several studies have found that they increase the risk of severe infections [2], others have not [3]. As a result, there remains ongoing controversy regarding the infection risk in patients on biological DMARDs (bDMARDs) [3-5]. In our descriptive review, our aim is to explore the infection risk with the use of bDMARDs. Additionally, we aim to explore current guidelines to help reduce the risk of infection while initiating bDMARDs in rheumatology patients. 

## Review

What are the different biological agents available for rheumatology patients?

In 1999, Maini et al. demonstrated the effectiveness of the biological agent infliximab, a tumor necrosis factor inhibitor (TNFi). The study showed that treatment with methotrexate plus infliximab was more effective in decreasing disease severity as compared to methotrexate monotherapy in the treatment of patients with active rheumatoid arthritis, who were not responding to methotrexate in the past [2]. 

Since then, several different bDMARDs have been approved for the management of different rheumatologic conditions. In contrast to conventional DMARDs (cDMARDs) which broadly suppress the immune system, bDMARDs target specific points in the inflammatory pathway, such as tumor necrosis factor (TNF), Janus kinase (JAK), B cells, and different interleukins (e.g., IL-1 and IL-6). Examples of these bDMARDs are listed in Table 1 [1].

**Table 1:** Different classes of biological agents used in the treatment of autoimmune conditions TNFi: tumor necrosis factor inhibitors

Drug class	Mechanism of action	Examples
TNFi	These inhibit tumor necrosis factor by directly binding to it or by binding to its receptor	Infliximab, etanercept, adalimumab, certolizumab
Janus kinase inhibitors	Inhibition of Janus kinases leads to inhibition of various cytokines, thereby decreasing inflammation	Tofacitinib, baricitinib, upadacitinib
B-cell inhibitors	These drugs bind to the CD20 receptor on B cells, thus preventing their activation	Rituximab
Interleukin inhibitors	These drugs target different interleukins involved in the inflammatory process	Tocilizumab (inhibits IL-6 receptors) and anakinra (inhibits IL-1 receptor)
T-cell activation inhibitors	These drugs interfere with synergistic signals that are essential for the activation of T-cells	Abatacept

In addition to those previously mentioned, newer drugs are being investigated and approved for the management of different rheumatological conditions. Most recently, results from phase 1b studies of rezpegaldesleukin, an IL-2 receptor agonist that enhances the activity of regulatory T cells (Tregs), showed improved disease outcomes in patients with rheumatologic skin diseases such as atopic dermatitis and psoriasis [6].

With the introduction of these new agents, rheumatologists have to be aware of the potential side effects and risk profiles of these drugs, in order to minimize harm and provide the best care to their patients. One key issue with bDMARD therapy in rheumatology patients is the increased infection risk, especially for those who are already exposed to prednisolone and cDMARDs [3,4].

What is the infection risk in bDMARDs compared with cDMARDs?

In the past years, there has been increasing evidence demonstrating increased risk of infection related to the use of biological agents. Several approaches have been made to quantify this risk; however, it remains to be a topic of controversy that requires further study. 

Most of the research has been focused on the use of biologics in the treatment of refractory rheumatoid arthritis. A meta-analysis demonstrated a twofold increased risk of serious infection requiring anti-microbial and/or hospitalization with TNFi compared to placebo [5]. Another meta-analysis published by Leombruno et al. in 2009 that extrapolated data from 18 randomized controlled trials showed no increase in the risk of infection with recommended doses of anti-TNF therapy [3]. Of note, this study had an overall limited follow-up period of less than one year. On the contrary, another meta-analysis published by Singh et al. in 2015 showed that standard doses of biological agents are associated with an increased risk of serious infections as compared to cDMARDs [4]. The study by Singh et al. included a significantly larger patient population of 42,000 subjects, compared to Leombruno et al., which had approximately 8000 subjects. In the study by Singh et al., the increased risk of infection was statistically significant only in the group that had been exposed to methotrexate in the past, compared to methotrexate-naive patients [4]. However, this is by far the largest study performed investigating the infectious risk in biological agents. Despite the difference in findings, both meta-analyses concluded that high-dose bDMARD therapy is associated with an increased risk of serious infections. The meta-analysis by Singh et al. [4] showed that compared to cDMARDs, standard-dose biological agents increased the risk of serious infection by 30% and high-dose biological agents increased the risk of infection by 90%. This demonstrates a dose-dependent increased risk of infections with higher doses of biological agents and a reduced risk with low-dose biologics. Increased risk of infections with biologics is further seen in a study by Chiu and Chen in 2020 which demonstrated that although both cDMARDs and bDMARDs increase the risk of bacterial infection, the associated risk with bDMARDs is significantly higher [7]. This study also showed that not all bDMARDs have equal risks of infection, with TNFi showing the highest increased risk of infection.

Aside from those involving rheumatoid arthritis, studies involving bDMARDs versus cDMARDs in treating other rheumatologic diseases have also exhibited an increase in infection risks overall. A systematic review of clinical trials showed a moderate increase in global infections in patients with spondyloarthritis [8], while another review revealed an 18% increase in the risk of overall infections in patients with psoriatic arthritis [9] with the use of biological agents.

What other factors determine overall infection risk?

There are a number of factors that contribute to the overall risk of infection in patients receiving bDMARD therapy. Evidence from previous studies have shown that the following factors contribute to overall infection risk: age, dosage, concomitant use, and time. There is limited data regarding the relative significance of each factor. The individual risk for each patient will be different, and each of these factors needs to be taken into consideration while assessing a patient's infection risk.

Age

In comparison to younger patients, the older population is more susceptible to infectious diseases due to a number of pathophysiologic and clinical factors such as immunosenescence and malnutrition [10]. 

Díaz-Lagares et al. in 2011 found that older age is an independent and significant predictive factor for serious infection, increasing the risk by 2.6% with the addition of biological agents [11]. However, this study did not adjust for the use of concomitant immunosuppressive agents, which may increase the risk of infection such as prednisolone. A study published in 2015 in a cohort of patients with inflammatory bowel disease (IBD) compared complications and cessation rates between younger and older patients (>60 years) on TNFi. A higher proportion of older patients had infectious complications in this study. They also demonstrated a higher discontinuation rate in this group compared to the younger patients [12]. In 2011, Galloway et al. published a study that investigated age as a risk factor for serious infection. Although this study did not demonstrate a significantly elevated relative risk of serious infection in the elderly, after adjusting for the use of concomitant immunosuppression (steroids and methotrexate), the absolute risk of infection remains higher in this group [13]. This may highlight the difference in clinical practice when treating elderly patients, with a tendency to use steroids in the management of their inflammatory arthritis rather than steroid-sparing agents, which in turn increases their infection risk. 

Dosage

Multiple studies have demonstrated a dose-dependent relationship between biological agents and infection risk, with a significantly higher risk with high doses of the medication. As previously mentioned, the meta-analysis by Singh et al. in 2015 showed a dose-dependent increase in infection risk, with higher doses demonstrating increased risk of infections and lower doses exhibiting reduced risk [4]. Meanwhile, Leombruno et al. analyzed 18 randomized trials and found that patients receiving high doses (around 2-3 times the recommended dose) of anti-TNF therapy had a twofold increased risk of serious infections [3]. Results from a study also showed that infection risk is especially high in patients receiving three or more courses of rituximab [11]. The dose-dependent association with infection risk appears to be relatively well established. Hence, for patients requiring the addition of a biological agent, the minimally effective dose should be maintained if possible.

Concomitant Use and Previous Treatment

Concomitant and previous use of conventional DMARDs have been demonstrated to increase the risk of infection in patients on bDMARDs [4]. Combining two or more bDMARDs increases the overall risk of serious infection, with an almost threefold increase as demonstrated by a meta-analysis published in 2018 [14]. In terms of infection risk when combining a bDMARD with a cDMARD, Singh et al. showed an increased risk with combination therapy [4], although results from a meta-analysis were not consistent with such finding as it showed no increased risk of infection with methotrexate and bDMARD combination compared with monotherapy with bDMARD only [15]. The overall infection risk is definitely higher if two or more bDMARDs are combined. It appears to be relatively safe to combine a cDMARD with a bDMARD in terms of infective risk, although caution should be exercised if patients are on concomitant steroids [16]. 

Time

Time remains a significant factor in the development of infections. Reviews done regarding the risk of infection related to TNFi treatment in rheumatoid arthritis have revealed an initial increased risk of infections, with the risk decreasing over time [17]. Askling and Dixon in 2008 concluded that shortly after treatment initiation, there is an observed increase in infection risk [18]. Askling et al. in 2007 found that in the first year of treatment, there was a 1.43 relative risk of infection, during the second year, the relative risk decreased to 1.15, and after the second year, the relative risk further decreased to 0.8213 [17]. Infection risk has been observed to be the highest during the first six months of treatment initiation [11,13]. It is hypothesized that this observed increase and then decrease in infection risk over time are likely due to a healthy user effect, wherein individuals with serious infections will stop treatment with biological agents, thus depleting the number of susceptible individuals [5]. Meanwhile, the initial increase in infection risk seen in most meta-analyses is likely because randomized trials focus primarily on the early phases of treatment initiation, especially in the first six months. Hence, clinicals need to be vigilant regarding infections during the initiation phase of bDMARDs more than the continuation phase. 

Which agents are more likely to cause infection?

Despite the overall evidence of increased infection risk, different biological agents seem to exhibit varying degrees of associated risk. It was observed that the risk of infection was increased by treatment with anti-TNF agents [5,19]. A research done on the off-label use of biological agents on systemic autoimmune diseases showed that rates of infection were dependent on which biological agent was used [11]. This study included rituximab, adalimumab, etanercept, and infliximab. Among those used in the study, the highest risk was seen with the use of infliximab, while etanercept exhibited the lowest risk of infection [11]. Another study that included etanercept, tocilizumab, and certolizumab found that when compared to etanercept and tocilizumab, certolizumab showed the lowest risk for serious infections in patients being treated for rheumatoid arthritis [20]. This study is comparable to another review of the German Biologics Register which found that there is a higher relative risk of infection with tocilizumab use as compared to those being treated with anti-TNF therapy [21]. A possible connection can be made as in the ADACTA trials, it was found that tocilizumab had superior efficacy compared to adalimumab [22]. This raises the possibility that tocilizumab may be a more potent drug, thus also leading to higher infection rates with increased suppression of the immune system. 

Due to ethical considerations, there are limited randomized controlled trials performing direct comparisons between different biological agents. Real-world data shows a mild difference in the overall infection risk with these agents [11,20]; however, as discussed in the following section, certain agents predispose patients to certain infections.

Which infections are more likely in patients on bDMARDs?

Studies have shown that the most common infections associated with bDMARD use are respiratory tract infections [11,20]. Other common sites of infection identified are skin and soft tissue infections, bacteremia/sepsis, urinary tract infections, and gastrointestinal infections. A variety of bacterial and viral infections have been found to be associated with DMARD therapy, and most commonly reported are those on tuberculosis (TB), hepatitis B, and herpes zoster reactivation [23]. Death from serious infections is also seen to be higher for these patients in comparison to the general public, with a 10% 30-day mortality seen in a large cohort study of about 20,000 patients using data from the British Society for Rheumatology Biologics Register for Rheumatoid Arthritis (BSrBr-rA) [20]. The most common pathogens associated with the use of bDMARDs are listed in Table 2.

**Table 2:** Common infections observed in patients on bDMARDs bDMARDs: biological disease-modifying anti-rheumatic drugs

Bacterial infections	Viral infections
Streptococcus pneumoniae	Hepatitis B
Staphylococcus aureus	Herpes zoster
*Listeria *spp.	
*Salmonella *spp.	
Mycobacterium tuberculosis	

Bacterial Infections 

Pathogens commonly implicated in bacterial infections in bDMARD patients were *Streptococcus pneumoniae *and *Staphylococcus aureus *[11]. These pathogens are known to cause respiratory infections. Additionally, intracellular bacterial species such as *Listeria*, *Salmonella*, and most specifically *Mycobacterium tuberculosis *infections were observed to occur more frequently in anti-TNF cohorts [24,25]. The risk of latent TB infection was also found to be higher in anti-TNF agents compared to IL-17 and IL-23 inhibitors [26]. This increased risk of latent TB reactivation may be explained by the role of TNF-alpha in maintaining granulomas which wall off the infection. This risk has also been recognized by the United States Centers for Disease Control and Prevention (CDC) after 12 cases of TB reactivation within 20 months between 2002 and 2003 were recorded [27]. Comparably, a study involving Swedish patients also exhibited a fourfold risk of latent TB reactivation in patients receiving anti-TNF therapy [28]. Reactivation of latent TB cases has been reported with different anti-TNF agents including infliximab [29], etanercept [28,30], and adalimumab [30].

Viral Infections 

Biological agents, particularly TNFi, also increase the risk of reactivation of viral infections such as hepatitis B. Lee and colleagues found that TNFi present a significant risk of hepatitis B reactivation, particularly in hepatitis B surface antigen (HBsAg)-positive patients [31]. In this study, the researchers also observed hepatitis B reactivation in hepatitis B occult carriers (patients who are HBsAg negative but hepatitis B core antibody (HBcAb) positive). However, the risk of hepatitis B reactivation appears to be lower in patients receiving antiviral therapy [32]. This increased risk of reactivation with TNFi use may be explained by the role of TNF in the proliferation of hepatitis B virus (HBV)-specific T cells that suppress HBV replication [33].

Another infection found to have an increased risk of reactivation associated with the use of bDMARDs is herpes zoster [34]. In 2009, Strangfeld et al. concluded that treatment with anti-TNF-alpha antibodies may be associated with an increased risk of herpes zoster [35] when compared to cDMARDs. The risk of herpes zoster was also observed to be significantly higher in patients on bDMARD therapy, particularly patients on JAK inhibitors such as tofacitinib [36-38]. Similarly, a large cohort study involving 34,000 patients in the Korean Health Insurance Review and Assessment Service database showed a significant increase in herpes zoster risk [39]. The risk of incident and recurrent herpes zoster was higher after tofacitinib treatment than other bDMARDs [39]. This increased risk of herpes zoster associated with JAK inhibitor use may be explained by the downregulation of antiviral immunity in keratinocytes and the reduction of T-cell activation seen after exposure to JAK inhibitors [40].

What are the recommended measures to reduce the risk of infection in patients on bDMARDs?

Due to the increased risk of infections, the European Alliance of Associations for Rheumatology (EULAR) recommends individualized shared decision-making with regard to immunization in patients on bDMARDs [41]. The 2021 American College of Rheumatology (ACR) recommends the addition of cDMARDs as an additional therapy rather than bDMARD in patients with moderate to high disease activity if they have had a serious infection within the last 12 months to reduce the infection risk [42]. 

There are a few considerations prior to commencing therapy for each patient.

First is a pre-biologic screen. An infection screen prior to commencing therapy, referred to as a pre-biologic screen, will help determine susceptibility to certain infections. This refers to performing serologies for each patient prior to commencing biological agents. There are no certain guidelines regarding which serologies to perform at this stage. Clinicians should consider screening for TB, human immunodeficiency virus (HIV), viral hepatitis, varicella zoster virus (VZV), and/or *Strongyloides*; however, the decision needs to be individualized based on certain risk profiles such as recent travel, country of origin/birth, and/or exposure history. The 2022 update from EULAR recommended latent TB infection screening in patients initiating bDMARD therapy [43]. Various studies have also recommended screening for latent TB before beginning treatment [44-46]. Screening can be done via the tuberculin skin test or QuantiFERON-TB Gold (QFT) test, and standards can differ depending on the region. 

Second is vaccination. The 2019 EULAR update recommends vaccination against certain infections (e.g., influenza, pneumococcal, tetanus, hepatitis A and B, and herpes zoster) in adult patients with autoimmune inflammatory rheumatic diseases (AIIRD) [41]. Meanwhile, yellow fever vaccination is recommended to be generally avoided by AIIRD patients [41]. 

Third is prophylaxis. Patients who are non-immune to VZV should receive advice regarding post-exposure prophylaxis if they are exposed to the virus [43]. Prophylaxis against *Pneumocystis jirovecii* pneumonia (PJP) should be considered in patients who are on bDMARDs with high-dose glucocorticoids [43].

Increased risk of malignancy 

Current evidence suggests a possible association between the use of bDMARDs and the risk of malignancy in rheumatology patients, especially in patients with rheumatoid arthritis. 

TNFi has been found to increase the likelihood of malignancy in multiple studies. In 2006, Bongartz et al. demonstrated that the use of TNFi (infliximab and adalimumab) increases the risk of malignancy in rheumatology patients with an odds ratio of 3.3 [5]. This study also demonstrated a dose-dependent increase in malignancy risk on anti-TNF therapy. With regard to non-TNFi bDMARDs, abatacept exposure has been observed to increase cancer risk as compared to cDMARDs [47]. 

However, rituximab or tocilizumab showed no significant increase in cancer risk compared to cDMARDs and TNFi [47]. On the other hand, JAK inhibitors have been shown to increase malignancy risk when compared to TNFi [48]. In 2021, the US Food and Drug Administration (USFDA) published results from initial safety trials of a JAK inhibitor, tofacitinib, which contained warnings on cardiovascular risk and malignancy risk of tofacitinib in contrast to TNFi [49]. Findings of the clinical trials were published the following year [38]. With increasing evidence of malignancy risk associated with bDMARDs, especially the JAK inhibitor tofacitinib, the EULAR has updated its recommendations in 2022 [43]. This update included the consideration of pertinent risk factors before the addition and initiation of JAK inhibitor therapy. This recommendation takes into account the cardiovascular risk and increased rates of malignancy associated with tofacitinib as opposed to other biological agents. A more recent study that aims to further elucidate the association of JAK inhibitors and cancer risk has reported evidence of an increase in the risk of non-melanoma skin cancer in individuals initiating JAK inhibitor therapy [50]. 

## Conclusions

The benefits of bDMARD therapy should be weighed against the risk of adverse events, especially the risk of infection and malignancy. Specific patient factors such as age, concomitant immunosuppression, and dose of the biological agent need to be considered, especially for patients on TNFi. To reduce the risk of subsequent infection, we recommend performing a pre-biologic screen and organizing appropriate vaccinations prior to commencing bDMARDs. 

As newer biological and targeted treatments are further developed, challenges with increased infection risk will continue. The expanding pool of evidence and studies should ultimately lead to the creation of personalized estimates of risk versus benefit analyses for each biological agent with the aim of providing the best care for each patient.
